# The effect of paraspinal muscle on functional status and recovery in patients with lumbar spinal stenosis

**DOI:** 10.1186/s13018-020-01751-1

**Published:** 2020-06-23

**Authors:** Wei Wang, Zhuoran Sun, Weishi Li, Zhongqiang Chen

**Affiliations:** 1 Department of Orthopaedics, Peking University Third Hospital, No. 49 North Garden Road, Haidian District, Beijing, 100191 China; 2 Peking University Health Science Center, No. 38 Xueyuan Road, Haidian District, Beijing, 100191 China

**Keywords:** Paraspinal muscle, Multifidus, Fatty infiltration, Cross-sectional area, Functional status and recovery

## Abstract

**Purpose:**

To investigate the association of paraspinal muscle quantity and quality with functional status in patients with lumbar spinal stenosis (LSS) and explore whether degeneration of paraspinal muscle could predict patients’ functional recovery.

**Methods:**

The data of 69 patients (26 males, 43 females; mean age 60.6 ± 9.4 years) with LSS was reviewed. The total cross-sectional area (tCSA), functional cross-sectional area (fCSA), and fatty infiltration (FI) of paraspinal muscle were measured. The Oswestry Disability Index (ODI) scores were used to reflect patients’ functional status. Correlations between measurements of paraspinal muscle and ODI scores were investigated by the Pearson correlation analysis. The multiple linear regression analysis was used to explore the correlation between change of ODI and other potential influence factors. Receiver operating characteristic curve was used to find out the most optimum cut-off value for predicting functional recovery.

**Results:**

The pre-operation ODI was significantly associated with multifidus muscle (MF) fCSA (*r* = − 0.304, *p* = 0.012), while the post-operation ODI was significantly correlated to MF FI (*r* = 0.407, *p* < 0.01). Preoperative MF FI was an independent influence factor for change of ODI. The best cut-off value of preoperative MF FI to predict improvement of functional status (change of ODI > 50%) was 33%.

**Conclusion:**

The preoperative degeneration of MF was significantly associated with patients’ functional status. Preoperative MF FI was a good predictor for assessing improvement of patients’ functional status. Evaluation of paraspinal muscle before operation could be helpful for surgeons to predict patients’ functional status and recovery.

## Background

The stability of the spine is dependent on the integrated function of the active, passive, and neural sub-systems [1]. As a composition of the active sub-system, the paraspinal muscle plays an important role in maintaining stability. The cross-sectional area (CSA) and fatty infiltration (FI) are two keys in evaluating the paraspinal muscles [2–6], which represent the quantity and quality of paraspinal muscles, respectively.

The atrophy of paraspinal muscles has been found in patients with chronic LBP and disc herniation [4, 7–9]. But only a few studies focused on the effect of paraspinal muscles on patients’ functional status [10, 11]. Fortin et al. found that greater multifidus muscle (MF) FI was associated with higher pre-operation Oswestry Disability Index (ODI) scores in patients with lumbar spinal stenosis (LSS) [10]. However, they ignored the postoperative functional status. Zotti et al. found that reduced MF CSA was associated with less favorable outcomes after surgery [11], but they only focused on MF. So it is unclear whether other paraspinal muscles are associated with patients’ functional status. Besides, it is also a question whether measurements of paraspinal muscles could predict the improvement of patients’ functional status.

This study aimed to investigate the association of paraspinal muscle quantity and quality with functional status in patients with LSS and explore whether degeneration of paraspinal muscle could predict the improvement of patients’ functional status.

## Methods

### Study design and inclusion criteria

This was a single-institution retrospective study approved by the relevant institutional Ethics Committee. Initially, a total of 72 patients with LSS who undertook lumbar fusion surgery from October 2018 to April 2019 were investigated. All of them presented with neurogenic claudication occasionally with concomitant radicular pain and failed with conservative therapy for at least 3 months before surgery. Three patients were excluded for the uncompleted follow-up data. Finally, 69 patients were included in this study. All patients were not engaged in special occupations, such as athletes, and they kept a normal lifestyle before the illness. The ODI scores were recorded. The other inclusion criteria of patients were as follows:

1. Older than 18 years
2. Without history of former spinal surgery
3. Without other spinal diseases except LSS
4. Had complete preoperative radiographic data
5. With a complete 6-month follow-up data

The exclusion criteria of patients were as follows:

1. With neuromuscular diseases
2. With hip joint or knee joint disease

The patients received preoperative education, general guidance after surgery, and instructions of trunk and abdominal muscle training. They underwent standardized procedures of surgery under general anesthesia, including pedicle screw fixation, bilateral laminotomy, and placing an interbody cage packed with autogenous bone. All surgeries were performed by three surgeons, who had more than 10 years of experience. The patients were encouraged to exercise by themselves in the hospital and at home. All patients could get out of bed in 3 days after surgery and exercised regularly after 3 weeks. No one needs a revision surgery in the last follow-up.

### Measurements of paraspinal muscle

Measurements of MF and erector spinae muscle (ES) were obtained from T2-weighted images by the Image J software. MRIs were required with Signa HDxt 3.0T (General Electric Company). The slice thickness was 3 mm with a 3-mm gap between each slice. Patients were placed in the supine position, with their legs straight and the lumbar spine in a neutral posture. Axial MRI was parallel to the inferior endplate of the vertebral body.

**Fig. 1:**
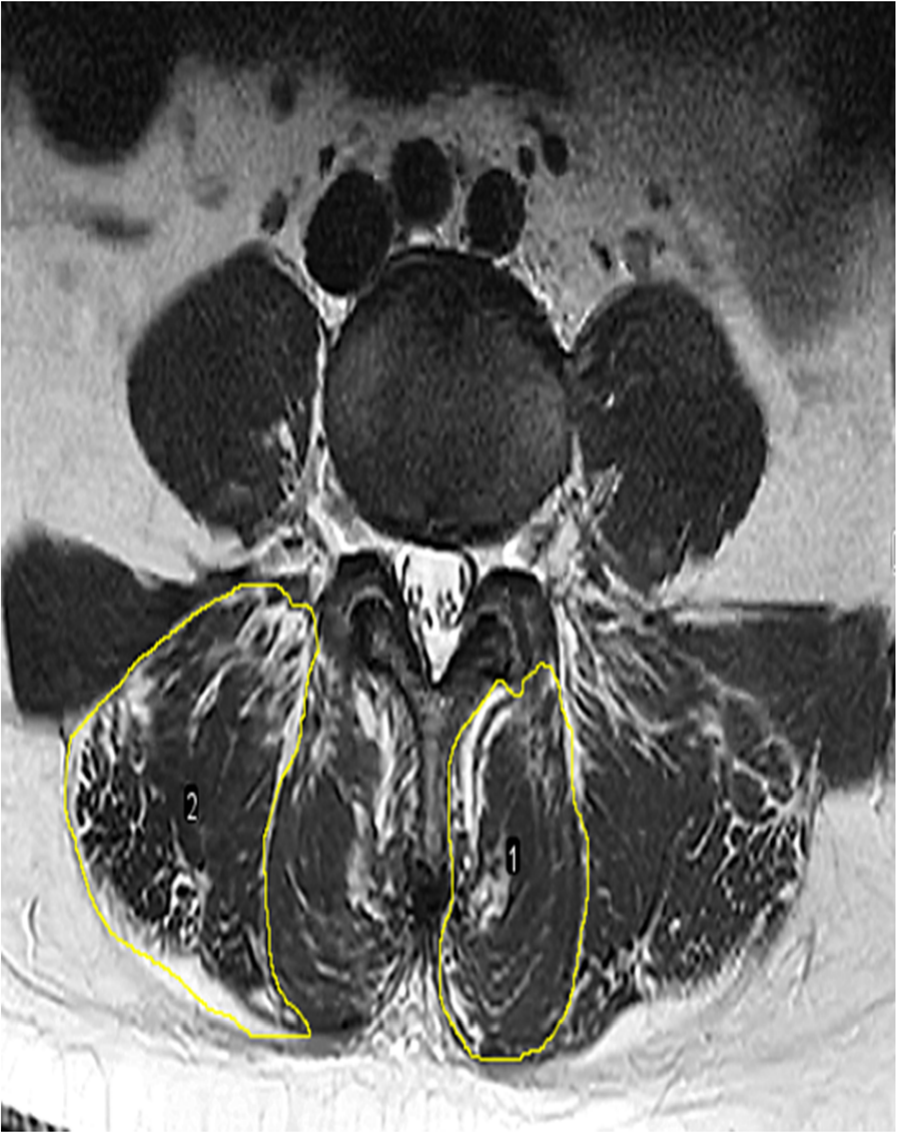
Measurement of total cross-sectional area (tCSA) for the multifidus (MF) and erector spinae muscle (ES)

**Fig. 2:**
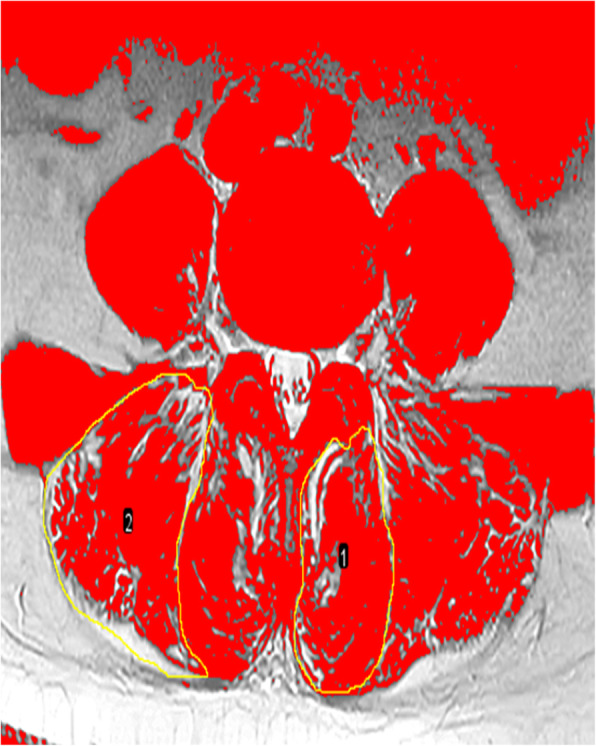
Thresholding technique to highlight lean muscle area and obtain the functional cross-sectional area (fCSA) of paraspinal muscles

All muscles were measured bilaterally at the inferior vertebral endplate of L4. The mean value of left and right paraspinal muscle was calculated. The parameters of muscle included total cross-sectional area (tCSA) (Fig. 1), functional cross-sectional area (fCSA, the area of lean muscle tissue), and fatty infiltration (FI, the ratio of tCSA minus fCSA to tCSA). The fCSA of muscle was measured by the thresholding technique (Fig. 2).

### Statistical analysis

SPSS version 17.0 was used to analyze the collected data. All values were expressed as mean ± standard deviation. The Pearson correlation analysis was also used to explore the relationship between paraspinal muscle parameters and other factors, such as age and ODI scores. Parameters between different genders were analyzed by the Mann–Whitney *U* test or independent-sample *t* test. The multiple linear regression analysis was performed to explore the influence factors for the change of ODI. We used the receiver operating characteristic (ROC) curve to find out the most optimum cut-off point of which presented the largest Youden index. Statistical significance was set at *p* value < 0.05.

## Results

### General data

There were 26 males and 43 females in this study. The average age of patients was 60.6 ± 9.4 years with a range from 32 to 77 years (Table 1). The average segment of fusion was 1.6 ± 0.7, which included L1–L2 (2), L1–L5 (1), L2–L3 (1), L2–L5 (5), L3–L4 (1), L3–L5 (27), and L4–L5 (32). The mean body mass index (BMI) was 26.6 ± 3.1 kg/m^2^. The average operation time was 126.9 ± 30.4 min. The mean blood loss during the operation was 318.7 ± 131.8 ml. The average hospital stay was 6.7 ± 1.1 days. The mean ODI scores were 55.5 ± 12.5 preoperatively, while the postoperative ODI scores were 26.7 ± 7.0 at 6-month follow-up. Change of ODI was defined as the ratio of preoperative ODI minus postoperative ODI to preoperative ODI. The mean change of ODI was 51.2 ± 9.6%. The mean and standard deviation of the different paraspinal muscle parameters are also presented in Table 1.

**Table 1:** Patients’ characteristics

	Overall	Male	Female	*p* value
Age (years)	60.6 ± 9.4	58.4 ± 8.8	61.8 ± 9.6	0.146
BMI (kg/m^2^)	26.6 ± 3.1	26.5 ± 2.1	26.7 ± 3.5	0.713
Number of operation segment	1.6 ± 0.7	1.7 ± 0.8	1.5 ± 0.6	0.662
Operation time (min)	126.9 ± 30.4	132.1 ± 31.6	123.8 ± 29.6	0.278
Blood loss (ml)	318.7 ± 131.8	326.9 ± 143.0	313.7 ± 126.0	0.853
Hospital stay (day)	6.7 ± 1.1	6.5 ± 1.2	6.8 ± 1.1	0.279
MF tCSA (mm^2^)	949.4 ± 199.8	1049.2 ± 225.3	889.0 ± 156.0	0.001**
ES tCSA (mm^2^)	1593.6 ± 361.5	1836.5 ± 341.2	1446.7 ± 289.3	< 0.001**
MF fCSA (mm^2^)	609.4 ± 183.8	718.7 ± 207.5	543.3 ± 131.1	< 0.001**
MF FI (%)	36.0 ± 11.4	31.6 ± 11.5	38.6 ± 10.5	0.011*
ES fCSA (mm^2^)	1082.1 ± 321.3	1331.5 ± 294.4	931.4 ± 231.9	< 0.001**
ES FI (%)	32.5 ± 9.9	27.7 ± 7.4	35.4 ± 10.1	0.001**
Pre-operation ODI	55.5 ± 12.5	51.9 ± 14.2	57.7 ± 11.0	0.061
Post-operation ODI	26.7 ± 7.0	24.7 ± 6.4	28.0 ± 7.1	0.055
Change of ODI (%)	51.2 ± 9.6	51.3 ± 9.9	51.1 ± 9.5	0.922

*BMI* body mass index, *MF* multifidus muscle, *ES* erector spinae muscles, *tCSA* total cross-sectional area, *fCSA* functional cross-sectional area, *FI* fatty infiltration

*p value < 0.05

**p value < 0.01

The value of age, BMI, number of operation segment, operation time, blood loss, and hospital stay did not have significant difference between different genders. The ODI scores in males were the same as those in females. The mean value of MF tCSA, ES tCSA, MF fCSA, and ES fCSA in males was 1049.2 ± 225.3, 1836.5 ± 341.2, 718.7 ± 207.5, and 1331.5 ± 294.4, respectively. The mean value of MF tCSA, ES tCSA, MF fCSA, and ES fCSA in females was 889.0 ± 156.0, 1446.7 ± 289.3, 543.3 ± 131.1, and 931.4 ± 231.9, respectively. The CSAs of paraspinal muscles were larger in males (*p* < 0.01). But the mean values of MF FI (*p* < 0.05) and ES FI (*p* < 0.01) in males were significantly smaller than females.

### Correlations between measurements of paraspinal muscle and ODI scores

The association of paraspinal muscle parameters with ODI scores and age was measured by the Pearson correlation analysis, and the results are recorded in Table 2. Not only pre-operation ODI but also post-operation ODI had a negative correlation with MF fCSA, which showed patients with severe degeneration of MF had worse functional status. Both MF FI and ES FI were associated with pre-operation and post-operation ODI. Patients with higher ODI scores had significantly higher MF FI and ES FI. Besides, age had a positive correlation with the MF FI (*p* < 0.01) and ES FI (*p* < 0.01), while it was negatively associated with MF fCSA (*p* < 0.01).

**Table 2:** Correlations between paraspinal muscle parameters and ODI scores

	MF tCSA	ES tCSA	MF fCSA	MF FI	ES fCSA	ES FI
Pre-operation ODI	− 0.174	− 0.119	− 0.418**	0.423**	− 0.286*	0.370**
Post-operation ODI	− 0.005	− 0.010	− 0.391**	0.606**	− 0.224	0.438**
Change of ODI	− 0.218	− 0.135	0.007	− 0.300*	− 0.051	− 0.142
Age	− 0.048	− 0.029	− 0.341**	0.494**	− 0.217	0.426**

*MF* multifidus muscle, *ES* erector spinae muscles, *tCSA* total cross-sectional area, *fCSA* functional cross-sectional area, *FI* fatty infiltration, *ODI* The Oswestry Disability Index

*p value < 0.05

**p value < 0.01

In order to eliminate the other factors’ effect, we used partial correlation analysis to analyze the relationship between paraspinal muscle parameters and ODI scores. The pre-operation ODI had a significant association with MF fCSA (*r* = − 0.304, *p* = 0.012) by controlling age. There existed a significant correlation between the post-operation ODI and MF FI (*r* = 0.407, *p* < 0.01) by controlling age and pre-operation ODI.

We also found that change of ODI had a significant correlation with MF FI (*p* < 0.05). The change of ODI reflected the improvement of patients’ functional status. We used the multiple linear regression analysis to investigate how the factors including age, gender, BMI, and MF FI influenced the change of ODI. According to Table 3, MF FI was independently associated with the change of ODI. Patients with lower preoperative MF FI were likely to have higher change of ODI.

**Table 3:** Results of multiple linear regression analysis in influence factors for change of ODI

	Regression coefficient	Standardized coefficient	*p* value
Age	− 0.079	− 0.078	0.566
Gender	1.551	0.079	0.519
BMI	0.676	0.216	0.070
MF FI	− 0.242	− 0.286	0.042*
Constant	45.69	–	< 0.01

*BMI* body mass index, *MF* multifidus muscle, *FI* fatty infiltration

*p value < 0.05

**Fig. 3:**
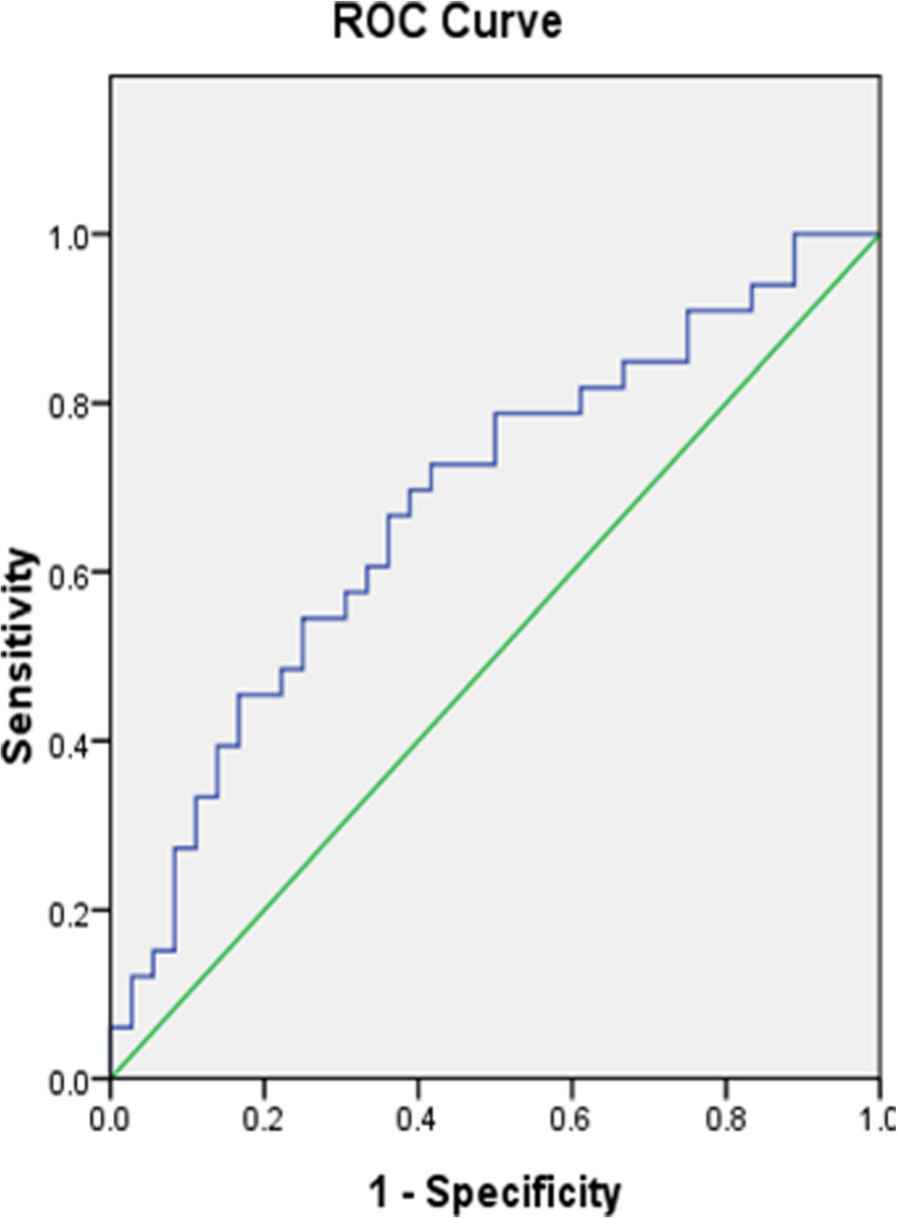
ROC curve to find the optimum cut-off point of MF FI to predict the change of ODI

The patients were divided into two groups (change of ODI ≤ 50% group and change of ODI > 50% group). We used ROC curves and calculated the Youden index to figure out the cut-off value of MF FI (Fig. 3). The best cut-off value of MF FI for predicting the change of ODI was 33% (AUC = 0.680, sensitivity = 0.727, specificity = 0.583). According to the best cut-off value of MF FI, patients were divided into two groups, respectively. For MF FI ≤ 33% group, the percentage of patients with change of ODI ≤ 50% was 32.3% (10/31), while it was 60.5% (23/38) for MF FI > 33% group.

## Discussion

This study demonstrated that smaller MF fCSA and higher MF FI on preoperative MRI scans were significantly associated with higher ODI scores, not only in pre-operation but also in post-operation. Similarly, preoperative ES FI was also positively correlated to ODI scores. The atrophy of paraspinal muscle was associated with patients’ functional status.

The relationship between paraspinal muscle and lumbar degeneration disease was of great interest in recent years [4, 11–13]. Some studies found atrophy of paraspinal muscle in patients with symptomatic LSS [14, 15]. But only few studies focused on the relationship between paraspinal muscle and functional status of patients [10, 11], and they did not fully assess paraspinal muscle or patient functional status. Fortin et al. found an association of greater MF FI with worse function in patients with LSS [10]. However, they did not focus on post-operation functional status. Zotti et al. found that reduced MF CSA was associated with less favorable outcomes after surgery [11], but they did not measure the quality of MF.

To further investigate the relationship between paraspinal muscle and functional status of patients, we measured tCSA, fCSA, and FI of MF as well as ES and explored all of the measurements’ association with patients’ functional status, not only in pre-operation but also in post-operation. The CSA and FI are two key parameters in evaluating the paraspinal muscles [2–6], which represent the quantity and quality of paraspinal muscles, respectively. Because a change in muscle composition can occur without a change in muscle size, fCSA is a better indicator of muscle atrophy and contractibility.

Age was an important factor for atrophy of paraspinal muscle and degeneration disease [2, 16]. Shahidi et al. found that lumbar muscle fat content was associated with age in individuals with lumbar spine pathology [2]. Takayama et al. also found that CSA of the paraspinal muscles decreased with age and fat infiltration increased with age [16]. Our results were consistent with them. Age attributed to the degeneration of paraspinal muscle.

Age was also associated with ODI scores. In order to eliminate age’s effect on the relationship between measurements of paraspinal muscle and ODI scores, we used partial correlation analysis. The results showed that atrophy of MF was independently associated with functional status. A study demonstrated that atrophy of paraspinal muscle was associated with muscle strength [17]. Ramos et al. found that stabilization exercises could improve lumbar multifidus fatigue in patients [18]. So exercises may be helpful for patients to improve their functional status.

Another important finding of this study was that preoperative MF FI could be a predictor for assessing patients’ functional recovery. When evaluating patients’ outcome, the change of ODI was also an important parameter, which reflected the improvement extent of patients’ functional status [11]. We explored the potential influence factors for the change of ODI, including age, gender, BMI, and preoperative MF FI. The result showed that preoperative MF FI was independently associated with the change of ODI. The change of ODI decreased with preoperative MF FI increasing. Preoperative MF FI could be a predictor for evaluating patients’ recovery. Chen et al. [19] also found that patients with MF FI < 25% had a greater reduction in ODI, which also supported our results.

The change of ODI > 40% was used as a clinically significant threshold in previous study [11]. The minimum clinically important percentage change in standardized outcome measures had been reported as 38–51% [20]. Considering the patients’ age in present investigation was younger than that in Zotti’s study, we used change of ODI > 50% as a clinically significant threshold. The MF FI ≤ 33% was the optimum cut-off value to predict the improvement of functional status.

In this research, we used the Image J software to measure paraspinal muscles. It was reliable in evaluating the measurements of paraspinal muscle [21, 22]. We measured paraspinal muscle at the level of L4 inferior vertebral endplate, which was the same as previous studies [10, 11, 15]. The effect of quality and quantity of paraspinal muscle on patients’ functional status was deeply explored in this study. But there were several limitations in this investigation. This was only a single-center retrospective study which might bring the selection bias. The sample size was relatively small. A prospective study with large participants is necessary to confirm the causal relationship.

## Conclusion

In this research, we found that the preoperative atrophy of MF was significantly associated with ODI scores, which reflected patients’ functional status. Among these measurements of paraspinal muscle, preoperative MF FI was a good predictor for assessing improvement of patients’ functional status. Evaluation of paraspinal muscle before operation could be helpful for surgeons to predict patients’ functional status and recovery.

## Data Availability

The data used and analyzed during the current study was available from the corresponding author on reasonable request.

## Abbreviations

LSS: Lumbar spinal stenosis
BMI: Body mass index
MF: Multifidus muscle
ES: Erector spinae muscles
tCSA: Total cross-sectional area
fCSA: Functional cross-sectional area
FI: Fatty infiltration
ODI: The Oswestry Disability Index
ROC: Receiver operating characteristic
